# Factors associated with insomnia and aggression among healthcare workers during the COVID-19 pandemic

**DOI:** 10.1192/j.eurpsy.2023.495

**Published:** 2023-07-19

**Authors:** D. Schneider-Matyka, A. M. Cybulska, K. Rachubińska, S. Grochans, A. Weymann

**Affiliations:** ^1^Department of Nursing; ^2^Department of Clinical Nursing; ^3^Student Scientific Club of Department of Nursing, Pomeranian Medical University, Szczecin, Poland

## Abstract

**Introduction:**

Healthcare workers are exposed to increased risks of insomnia and aggression during the COVID-19 pandemic.

**Objectives:**

The aim of the study was to estimate the prevalence rate of insomnia and aggression and identify associated risk factors among healthcare workers during the COVID-19 pandemic

**Methods:**

A total of 264 healthcare workers participated in the study. The study was conducted with the diagnostic survey method, using the Buss-Perry Aggression Questionnaire, the Athens Insomnia Scale, the Pittsburgh Sleep Quality Index, and a questionnaire of our authorship.

**Results:**

The vast majority of the respondents (81.06%) suffered from insomnia and had poor sleep quality (78.03%). Education (p=0.038), marital (p=0.043) and parental status (p=0.004), and contact with patients suffering from COVID-19 (p=0.024) were statistically significant contributors to insomnia. Working time was found to significantly correlate with insomnia (r=0.124 p=0.044) and a physical aggression (r=0.168 p=0.006), anger (r=0.121 p=0.05), a verbal aggression (r=-0.132 p=0.032). Age was found to significantly correlate with total aggression (r=-0.133 p=0.031), verbal aggression (r=-0.138 p=0.025), anger (r=-0.151 p=0.014). Sex was found to be statistically significantly related to physical aggression (p=0.017), anger (p=0.032), and hostility (p=0.002).

**Conclusions:**

A considerable proportion of HCWs experienced sleep disorders during the pandemic, emphasizing the need to establish ways to reduce long-term adverse outcomes associated with chronic insomnia and adjust interventions under pandemic conditions. Our findings confirm that insomnia and poor sleep quality are consistently associated with aggression.

**Disclosure of Interest:**

None Declared

